# Consensus design for improved thermostability of lipoxygenase from *Anabaena* sp. PCC 7120

**DOI:** 10.1186/s12896-018-0468-4

**Published:** 2018-09-20

**Authors:** Hui Qian, Chong Zhang, Zhaoxin Lu, Bingjie Xia, Xiaomei Bie, Haizhen Zhao, Fengxia Lu, Guang-Yu Yang

**Affiliations:** 1 College of Food Science and Technology, Nanjing Agricultural University, 1st Weigang, Nanjing, 210095 People’s Republic of China; 2 State Key Laboratory of Microbial Metabolism, College of Life Science and Biotechnology, Shanghai Jiao Tong University, 800 Dongchuan Rd, Shanghai, 200240 People’s Republic of China

**Keywords:** Lipoxygenase, Thermostability, Specific activity, Consensus concept

## Abstract

**Background:**

Lipoxygenase (LOX) from *Anabaena sp*. PCC 7120 (Ana-rLOX) offers important applications in the food industry, especially for improving aroma and dough rheological properties. However, industrial applications of LOXs have been limited by their poor thermostability. Herein, we report a bioinformatics-based consensus concept approach for the engineering of thermostable Ana-rLOX.

**Results:**

A series of mutations (N130D, G260A, S437T, N130D/G260Q, N130D/S437Y) showed higher thermostability and activity than the wild-type enzyme. Thus, N130D/G260Q exhibited a 6.6-fold increase in half-life and 2.45 °C increase in unfolding temperature; N130D/S437Y showed a 10 °C increase in optimal temperature. The secondary structure did not change much that contributed to improved thermostability were investigated in detail using circular dichroism. Homology modeling suggested that enhanced thermostability and specific activity may result from favorable hydrophobic interactions.

**Conclusions:**

A series of mutations were achieved, showing higher thermostability and activity than the wild-type enzyme by semi-rational mutagenesis with limited structure information. Our findings provide important new insights into molecular modifications aimed at improving Ana-rLOX thermostability and activity.

**Electronic supplementary material:**

The online version of this article (10.1186/s12896-018-0468-4) contains supplementary material, which is available to authorized users.

## Background

Lipoxygenases (LOXs; EC1.13.11.12) are a family of non-heme iron-containing dioxygenases that catalyze regio- and stereospecific dioxygenation of polyunsaturated fatty acids(PUFAs) containing a 1-cis,4-cis-pentadiene structure to form fatty acid hydroperoxides [1]. LOX-catalyzed oxidative coupling reactions are of interest to the food industry. They are applied in bread-making to bleach carotenoids and to improve dough rheology [2, 3]. In addition, LOX also played an important role in aroma compound formation. Because of their unique properties, LOXs have been widely utilized in the food, pharmaceutical [4, 5] and textile industries [6, 7]. However, industrial applications of LOXs have been relatively limited because of their poor thermostability, it is essential to generate LOXs with enhanced activity and thermostability.

Numerous protein engineering strategies have been attempted to improve protein stability through modification of various structural features [8]. Directed evolution, rational design, and semi-rational design methods have been used successfully to overcome certain limitations of enzymes and to improve thermostability [9–11]. Although some of these approaches have led to improving LOX thermostability [12], protein engineering aimed at strengthening thermostability and catalytic activity remains a challenging and exciting field [13]. This is particularly true when structural and mechanistic information about an enzyme is very limited, making it difficult to rationally predict the function of targeted amino acid residues [14]. “Consensus concept” is a rapidly developing theoretical method in protein engineering [15], which requires only protein family sequence information. Consensus concept assumes that the amino acid appearing most frequently among homologues at a specific position, contributes more than other residues at that position to enzyme stability. The method has been applied successfully to ameliorate protein thermostability [16–18].

In our previous studies, we cloned, expressed, and characterized the *Ana-rLOX* gene from *Anabaena* sp. PCC 7120, and this enzyme had also been successfully expressed in *Bacillus subtilis*(generally recommended as safe, GRAS) [19], so that Ana-rLOX could be used in food processing safely. The cloned enzyme (Ana-rLOX) had high specific activity but low stability, and was therefore unsuitable for many industrial applications [12]. Directed evolution is not a viable approach because there is no efficient high-throughput screening method for LOX [20]. In the present study, we employed sequence information about the LOX family to determine mutation sites on the basis of the consensus concept and constructed “small but smart” saturated mutant libraries for selection of Ana-rLOX mutant enzymes with enhanced thermostability. This approach allowed us to generate a series of mutants with greatly improved thermostability.

## Methods

### Materials

Ni^2+^-nitrilotriacetate (Ni-NTA) was purchased from Qiagen (Hilden, Germany). Linoleic acid was purchased from Sigma (Steinheim, Germany). PrimeSTAR DNA polymerase, enzyme *Dpn* I, and the genomic DNA purification kit were from TaKaRa (Dalian, China). Isopropyl β-D-l-thiogalactopyranoside (IPTG) and ampicillin were from Sangon Biotech (Shanghai, China). All compounds were of reagent grade or higher.

Cloning and expression strains were purchased from TaKaRa. *E. coli* BL21(DE3) and expression vector pET-32a were used for protein expression.

### Analysis of consensus amino acids

To identify key sites for mutagenesis, a simple approach is to align the target enzyme with its closely related homologs. Amino acid sequences common to the LOX family were downloaded from the pfam [21] and GenBank databases. Similarities between Ana-rLOX and LOX family proteins were analyzed using the Basic Local Alignment Search Tool (BLAST; NCBI). In contrast to other sequence-selection methods and because Ana-rLOX has low sequence identity with other LOX family proteins, we selected the 100-most similar LOX sequences. The selected sequences were aligned using ClustalX2, generating a phylogenetic tree by Neighbour Joining approach, which was then used to group the sequences into subsets. The most relevant sequences were chosen for analysis and were set to represent the main branches of the phylogenetic tree. The success of this approach depends on the phylogenetic diversity of the available sequence set [22]. The consensus sequence was calculated by ESpript [23] and either consensus positions or most abundant positions were determined. On the basis of the consensus result, non-consensus amino acids were substituted by consensus amino acids using site-directed saturation mutagenesis.

### Construction of saturation mutagenesis libraries

The plasmid pET-32a/*Ana-rLOX* encoding the LOX gene from *Anabaena* sp. PCC 7120 was used to construct the libraries [12], *Ana-rLOX* gene is 1365 bp. Mutants were prepared by the Quik Change® site-directed mutagenesis method [24] with primers containing NNK/MNN (K = G/T,M = A/C,N = A/C/G/T) degeneracy at target sites. Primers are listed in Additional file 1: Table S1.

Amplification was performed using the following temperature settings: 2 min at 98 °C; 30 cycles of 10 s at 98 °C, 15 s at 55 °C, and 7 min at 72 °C; final 10-min extension at 72 °C. PCR products were digested with *Dpn* I to remove the parent plasmid.

The created libraries were transformed into *E. coli* BL21(DE3) and selected for ampicillin resistance. Clones were picked in 96-well plates containing 200 μL Luria-Bertani (LB) medium with 100 μg/mL ampicillin. After growth at 37 °C overnight, the cultures were used to inoculate fresh medium in a new 96-well plate and incubated at 37 °C. IPTG was added to induce expression of the target protein at 16 °C. Due to the use of degenerate primers (NNK), each saturation mutagenesis library must have contained at least 200 colonies to overlay 20 kinds of amino acid codons. Screening was performed as described previously [12]. Selected positive clones were tested independently four more times. Confirmed positive clones were sequenced, and enzymes were expressed, purified, and characterized.

### Enzyme production and purification

For a detailed characterization of Ana-rLOX and its variants, *E. coli* BL21(DE3) transformed with expression plasmids was used. For enzyme production, 2% of overnight culture was inoculated in 100 mL LB medium containing 100 mg/mL ampicillin, and incubated at 37 °C with continuous shaking (180 rpm) until late logarithmic phase. Target protein expression was induced by addition of IPTG (final concentration 100 mg/mL). Protein induction was performed at 16 °C for 16 h. Cells were harvested by centrifugation (8000×*g*, 10 min, 4 °C), resuspended in 10 mL phosphate-buffered saline (PBS; 300 mM NaCl, 50 mM phosphate buffer, pH 8.0), and the obtained cell pellets were subjected to sonication. The crude extract was centrifuged as above to remove cell debris.

Crude enzyme was loaded into Ni-NTA resin to purify the protein, utilizing the His-tag encoded by pET-32a. SDS-PAGE analysis was performed on a 12% running gel [25], and resolved proteins were visualized by Coomassie Brilliant Blue G-250 staining. Protein concentration was measured by the Bradford method [26] with bovine serum albumin as the standard.

### Enzyme assays

Enzyme activity assays were performed as described previously [27]. Ana-rLOX activity was assayed at 30 °C using linoleic acid as substrate, and Tris-HCl (pH 9.0) as buffer. One unit of activity was defined as the quantity of enzyme required to synthesize 1 μM hydroperoxide, with molar extinction coefficient (ε) = 25,000 L/(mol·cm) [28].

Hydroperoxidation was determined at 234 nm in a spectrophotometer (UV-2450; Shimadzu Co., Kyoto, Japan). Enzyme activity was calculated using the initial linear part of the curve.

### Enzyme stability

Kinetic stability was assessed by incubating the purified enzyme (2.0 mg/mL) in a 50 °C water bath with sampling at regular intervals. Residual activity was measured and compared to that of the untreated enzyme. Thermostability was evaluated at 50 °C. Half-life (t1/2) was calculated after fitting experimental data to the equation ln Y = −kdX + b, assuming first order inactivation (t1/2 = ln 2/ kd). To construct the melting curves and determine the melting temperature (Tm), samples were heated at 1 °C per min from 20 to 90 °C and the circular dichroism (CD) ellipticity signal at 222 nm, which showed the maximal change with temperature, was monitored.

### Circular dichroism analysis

CD analysis was performed using a spectropolarimeter (model J-810, Jasco, Tokyo, Japan). Enzyme samples were dissolved in 10 mM PBS, pH 7.4, at a concentration of 0.1–0.15 mg/mL and loaded on a quartz cuvette (path length 1 cm). The spectrum of a buffer blank was subtracted. Helical and sheet contents of proteins were estimated using the online program DICHROWEB [29].

### Homology modeling

To analyze the mutated residues, the structures of Ana-rLOX and its mutants were simulated by Phyre2 software [30]. Lipoxigenase from *Cyanothece sp.* PCC 8801 (PDB:5EK8) [31] was used as template.

## Results

### Mutation design guided by the consensus concept

**Fig. 1:**
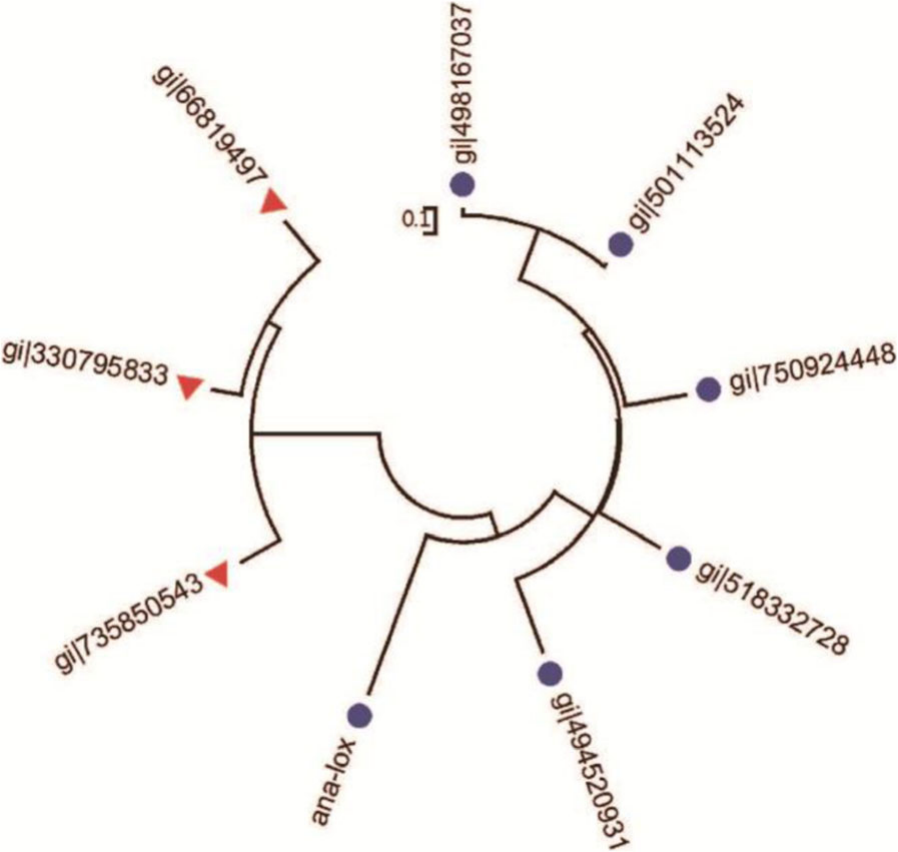
Phylogenetic tree of LOX enzymes. These sequences were chosen for multiple sequence alignment

The consensus concept is a bioinformatics-based protein design method that replaces specific amino acids with the amino acid that most frequently occurs at that position in the protein family [32, 33]. The amino acid sequence of Ana-rLOX was used to identify homologous sequences in the GenBank database. Sequence identity is critical to successfully determine the consensus sequence. Sequences with a close phylogenetic relationship with Ana-rLOX were selected, but those with > 95% identity were removed to avoid bias caused by repetition of similar sequences [8]. Therefore, sequences with identities between 30 to 90% were chosen for multiple sequence alignment (Fig. 1), and most of the stable amino acids in evolution were selected.

**Fig. 2:**
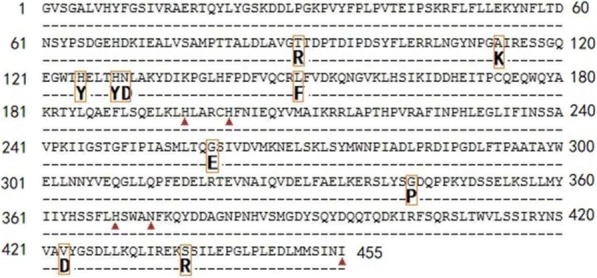
Protein sequence of Ana-rLOX. Ten positions indicating in bold were left for constructing saturated mutagenesis libraries,and metal liganding amino acids were labeled by triangle()

To reduce number of the candidated amino acids and improve quality of the mutagenesis libraries, homologous model of Ana-rLOX was utilized in selecting mutation sites. First, residues within 5 Å of the active site were excluded to avoid compromising enzyme activity. Second, mutations that would introduce hydrophobic amino acids on the protein surface or hydrophilic amino acids in the protein core were discarded. Third, mutations that would destroy existing hydrogen-bonds, salt-bridges, or other stabilizing interactions were also avoided. Consequently, ten positions was left for constructing saturation mutagenesis libraries with which to further investigate changes in thermal stability (Fig. 2).

### Characterization of mutants with improved thermostability

The ten selected residues were subjected to saturation mutagenesis and screening revealed several mutants with improved thermostability. Combinational mutagenesis experiments were performed in the next round by selecting the best mutant, N130D, as template; N130D/S437Y and N130D/G260Q were identified as providing further stability enhancement.

All the above mutants presented higher optimum temperatures (Table 1). For example, mutants S437T and N130D/S437Y showed highest activity at 45 °C, which is 10 °C higher than the wild-type enzyme, and thus may be suitable for industrial applications at such temperatures. At the same time, the half-lives (t_1/2_^50°C^) defined by the temperature at which the enzyme loses 50% of activity, increased from 2.3 min for wild-type to 9.3 min and 11.0 min for S437T and N130D/S437Y, respectively. The most thermostable combinational mutant, N130D/G260Q, exhibited a 6.6-fold longer half-life than the wild-type. T_m_ values determined by CD spectroscopy (Additional file 1: Figure S2), revealed a modest increase of 1.4 °C in mutant N130D compared to wild-type. To further increase thermostability, we tried to combine this mutation with S437Y and G260Q and obtained increases of 2.1–2.5 °C. These results indicate that combinations of beneficial amino acid substitutions correlated with significant structural changes, which contributed to improved thermostability.

**Table 1:** Thermal stability of mutants

Enzyme	Optimal temperature (°C)	Half-life(t_1/2_^50°C^) (min)	T_m_ (°C)
WT	35	2.3 ± 0.1	48.17
G260A	35	9.2 ± 0.4	48.61
S437T	45	9.3 ± 0.5	49.42
N130D	40	10.2 ± 0.4	49.59
N130D/S437Y	45	11.0 ± 0.5	50.28
N130D/G260Q	40	15.2 ± 0.9	50.62

In addition to improved thermostability, all mutant enzymes exhibited ameliorations in specific activities (Table 2). The specific activity of mutants S437T and N130D/G260Q was 1.7 and 2-fold higher than wild-type, respectively. This finding indicates that both thermostability and specific activity can be improved at the same time by the consensus method.

**Table 2:** Characterization of mutations

Enzyme	Specific activity (IU/mg)	*K*_m_ (μmol/L)	*k*_cat_ (1/s)	*k*_cat_/K_m_ L/(mmol·s)
WT	10.4 ± 0.2	56 ± 4.0	30.3 ± 1.6	541.1
G260A	22.5 ± 0.8	34 ± 2.3	51.7 ± 2.5	1520.6
S437T	27.6 ± 0.8	56 ± 4.7	117.6 ± 6.4	2100.0
N130D	22.7 ± 0.5	30 ± 2.6	57.7 ± 1.8	1923.3
N130D/S437Y	15.2 ± 0.9	15 ± 1.1	31.8 ± 1.9	2120.0
N130D/G260Q	21.0 ± 0.6	13 ± 1.0	35.4 ± 2.6	2724.6

To compare the catalytic reactions between wild-type and mutants, kinetic parameters were determined (Table 2). S437T displayed no changes in *K*_m_ and an increase of 288% in *k*_cat_ compared to wild-type, indicating that the mutant did not change its binding affinity for the substrate and had higher specific activity. Interestingly, the two double mutants N130D/S437Y and N130D/G260Q exhibited a 73% and 77% decrease in *K*_m_, respectively, indicating higher relative affinity for the substrate, and a consequent 290–400% increase in catalytic efficiency (*k*_cat_/ *K*_m_). This result indicates that enhanced thermal tolerance was likely accompanied by improved catalytic activity.

### Structural analysis of the mutations

The CD spectra of the thermostable variants were analyzed to determine whether the mutations affected the protein’s secondary structure (Additional file 1: Figure S2). Results showed that when the mutation was introduced at the N130 position, the secondary structure changed almostly nothing. When mutation S437T or G260Q was introduced, the β-sheet content increased to 12.4% and 16.1%, respectively (Table 3), revealing that the combing mutations influenced the secondary structure of the enzyme more than one point mutations.

**Table 3:** Secondary structure prediction by circular dichroism

Enzyme	Helix (%)	Sheet (%)	Turn (%)	Random (%)
WT	48.3	8.4	15	28.2
N130D	49.9	8	15.2	26.9
N130D/S437T	46.3	12.4	15.2	26.1
N130D/G260Q	42.5	16.1	12.7	28.7

**Fig. 3:**
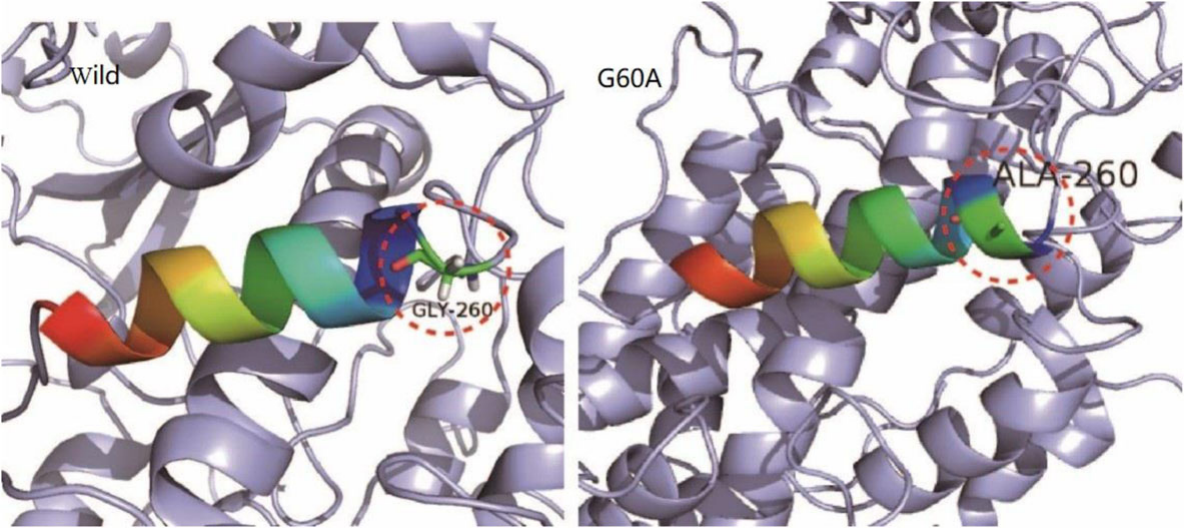
G260A substitution was located on the loop region of Ana-rLOX. Substitution of Gly with hydrophobic residue Ala at position 260 resulted in enhanced hydrophobic interactions

To gain deeper insight into the structural basis of thermal stability, a molecular model of the mutant was constructed. Substitution of Gly with hydrophobic residue Ala at position 260 resulted in enhanced hydrophobic interactions. The structure of G260A showed that position 260 was located in the surface loop region of the protein (Fig. 3). Stabilization of loops is known to play an important role in protein stability [34].

The substitution G260A has a similar effect as S437T, introducing one extra methyl group into Ana-rLOX that enhanced the hydrophobic interactions within the protein. Interestingly, the combinational mutants exhibited greater improvement when the mutation N130D was accompanied by substitutions G260Q and S437Y but not G260A or S437T.

## Discussion

Consensus concept is an appealing strategy for increasing the stability of proteins, as it relies only on sequence information [18] and, particularly, multiple sequences alignment. Nevertheless, even this method can only help determine the candidate residues to be mutated, but cannot predict the specific amino acid that could improve the enzyme. In this study, we investigated the difference between site-directed mutagenesis and saturation mutagenesis guided by the consensus concept. Half-lives of mutations N130D, S437T, and G260A were all significantly higher than in the wild-type (Table 4). We show that mutations introduced at G260 and S437 were not in accord with the consensus result, only mutation N130D was the “consensus amino acid”. We proposed that consensus approach could predict the position deserved to be mutated [16], and saturation mutagenesis was very efficient at improving the success rate of the experiment..

**Table 4:** Comparative effects of site-directed mutagenesis and saturation mutagenesis

Enzyme	Probability (%)	Half-life (t_1/2_^50°C^) (min)
N130D	55	10.2 ± 0.4
N130S	11	7.2 ± 0.3
G260E	55	4.7 ± 0.3
G260A	0	9.2 ± 0.4
S437R	55	< 2.3 ± 0.1
S437T	0	9.3 ± 0.5
S437K	0	6.4 ± 0.4

**Fig. 4:**
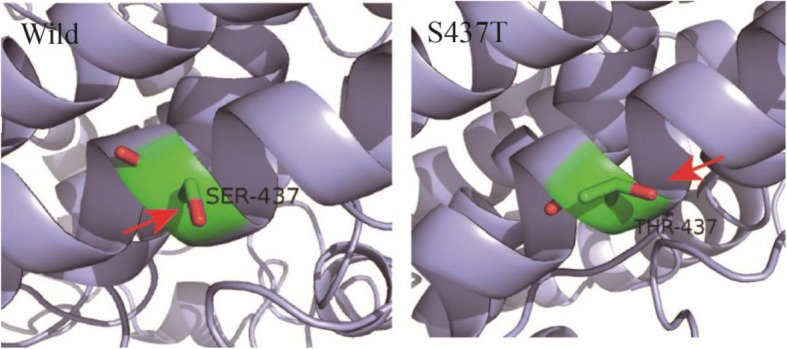
S437T mutation introduced one extra methyl group into Ana-rLOX, that enhanced the hydrophobic interactions within the protein

Three mutants (G260A, S437T, and N130D) produced on the basis of a structure-guided consensus method displaying varying degrees of improvements in thermostability (Table 2). The structure of G260A based on homology modeling showed that position 260 was located in the surface loop region of the protein (Fig. 3). Stabilization of loops, which plays an important role in protein stability, can be enhanced by engineering loop residues [35]. Substitution of Gly with Ala at position 260 resulted in enhanced hydrophobic interactions, promoting the formation of α-helix regions (Fig. 3), and improving stability of Ana-LOX through a more stable secondary structure. A effect was observed for S437T, whose half-life was also 4-fold higher than wild-type. Here, introduction of an extra methyl group into Ana-LOX which contributed increasing hydrophobic interaction (Fig. 4). These findings indicated us that enhanced enzyme thermostability depends mainly on favorable hydrophobic interactions.

Interestingly, all of our consensus mutations improved both thermostability and specific activity of Ana-LOX relative to wild-type. Although the consensus method has been found to be effective for increasing enzyme stability, such mutations are successful in only about 50% of cases [18]. As a matter of fact, thermostability and specific activity are partially independent properties, even though they can be optimized together in a given enzyme [36]. In a previous study using site-directed mutagenesis based on computer-aided rational design, the mutant V421A/V40A showed a 1.13-fold increase in thermostability and 80.07% increase in specific activity [37]. In the present study using consensus concept with saturation mutagenesis, the mutant N130D/G260Q showed a 6.6-fold increase in half-life at 50 °C and 2.0-fold increase in specific activity relative to Ana-rLOX. Thus, the consensus method proved efficient at screening Ana-rLOX mutants for enhanced thermostability and specific activity. Moreover, it allowed for significant increases in thermostability in comparison with alternative techniques such as rational design.

## Conclusion

Ana-rLOX mutants showing significant improvements in both thermostability and specific activity were produced by semi-rational mutagenesis based on sequence alignment and structural analysis. Mutant N130D/G260Q exhibited a 6.6-fold increase in half-life, and mutant N130D/S437Y showed a 10 °C increase in optimal temperature. Additionally, the catalytic efficiency (kcat/Km) was also improved. Our findings provide important new insights into molecular modifications aimed at improving Ana-LOX thermostability and activity.

## Additional file

Additional file 1: **Table S1.** Saturation mutagenesis primers. **Figure S1.** Purification of wild-type and mutants. M: Marker, 1:wild, 2: N130D, 3: G260A, 4: S437T, 5: N130D/S437Y, 6: N130D/G260Q. Crude enzyme was loaded onto Ni-NTA resin to purify the protein, utilizing the His-tag encoded by pET-32a. SDS-PAGE analysis was performed on a 12% running gel and were visualized by Coomassie Brilliant Blue G-250 staining. **Figure S2.** T_m_ values of enzymes determined by differential scanning calorimetry. **Figure S3.** Circular dichroism of wild-type and mutants. (DOC 320 kb)
