# Supplementary material for: Consensus design for improved thermostability of lipoxygenase from Anabaena sp. PCC 7120
Source: BMC Biotechnol. 2018 Sep 20;18:57. doi: 10.1186/s12896-018-0468-4 (PMC6148764; doi:10.1186/s12896-018-0468-4)

Additional file 1

Table S1 [Saturation](http://cn.bing.com/dict/clientsearch?mkt=zh-CN&setLang=zh&form=BDVEHC&q=饱和突变引物) [mutagenesis](http://cn.bing.com/dict/clientsearch?mkt=zh-CN&setLang=zh&form=BDVEHC&q=饱和突变引物) [primer](http://cn.bing.com/dict/clientsearch?mkt=zh-CN&setLang=zh&form=BDVEHC&q=饱和突变引物)s.

| Primers | Sequence（5’→3’） |
| --- | --- |
| L150-f | TCAATGTCGG**NNK**TTTGTTGATAAACAAAATG |
| L150-f | TATCAACAAA**MNN**CCGACATTGAACAAAATC |
| H125-f | AAGGCTGGACA**NNK**GAGCTTACTC |
| H125-r | AGTAAGCTC**MNN**TGTCCAGC CTTC |
| G260-f | GCTAACGCAG**NNK**TCTATTGTTGATGTAA |
| G260-r | AACAATAGA**MNN**CTGCGTTAGCATG |
| T90-f | CAGTTGGC**NNK**ACTGATCCCACC |
| T90-r | GGGATCAGT**MNN**GCCAACTGCCAA |
| S437-f | GGAAAAATCA**NNK**ATATTGGAACCAGGACTTC |
| S437-r | TCCAATAT**MNN**TGATTTTTCCCGAATGAGC |
| V423-f | AGTTGCA**NNK**TATGGATCGGATTTACTCAAAC |
| V423-r | GATCCATA**MNN**TGCAACTGA GTTGTATCGA AT |
| N130-f | TTACTCAC**NNK**CTGGCAAAATATGACATCAAG |
| N130-r | TTTGCCAG**MNN**GTGAGTAAGCTCATGTG |
| A113-f | AATCCAGGT**NNK**ATTCGCGAATCTT |
| A113-r | TTCGCGAAT**MNN**ACCTGGATTGTAC |
| H129-f | GCTTACT**NNK**AATCTGGCAAAATATGAC |
| H129-r | CAGATT**MNN**AGTAAGCTCATGTGTCCAG |
| G343-f | TCTTTACTCC**NNK**GACCAACCACC |
| G343-r | GGTTGGTC**MNN**GGAGTAAAG ACTTC |

Figure S1 Purification of wild-type and mutants

M: Marker, 1:wild, 2: N130D, 3: G260A, 4: S437T, 5: N130D/S437Y, 6: N130D/G260Q

Crude enzyme was loaded onto Ni-NTA resin to purify the protein, utilizing the His-tag encoded by pET-32a. SDS-PAGE analysis was performed on a 12% running gel and were visualized by Coomassie Brilliant Blue G-250 staining.


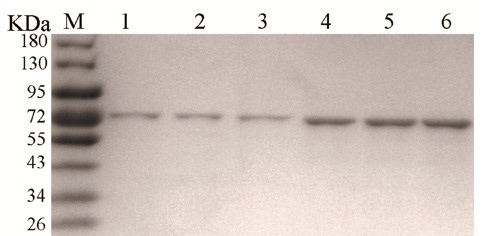


Figure S2 T_m_ values of enzymes determined by differential scanning calorimetry.


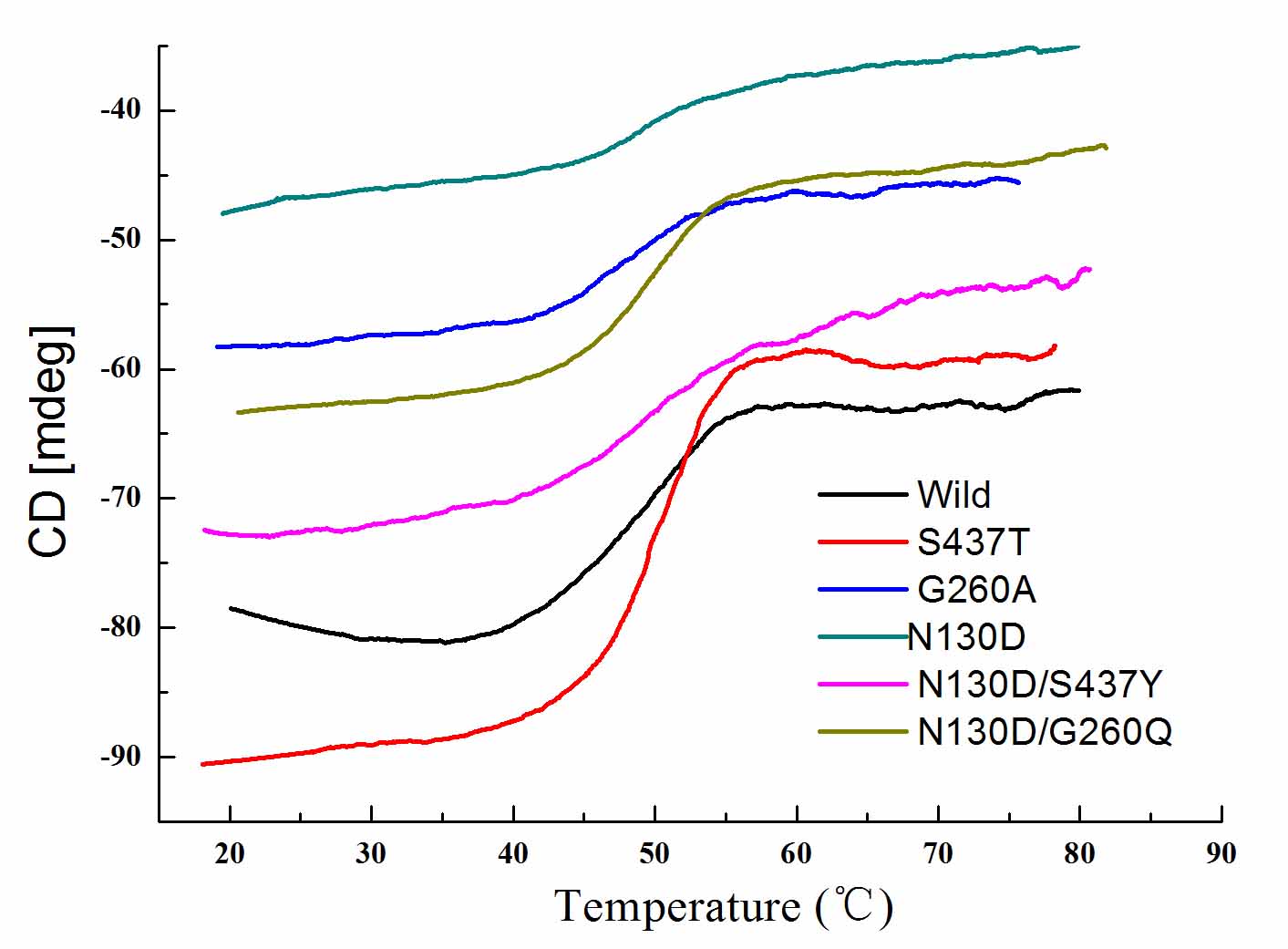


Figure S3 Circular dichroism of wild-type and mutants


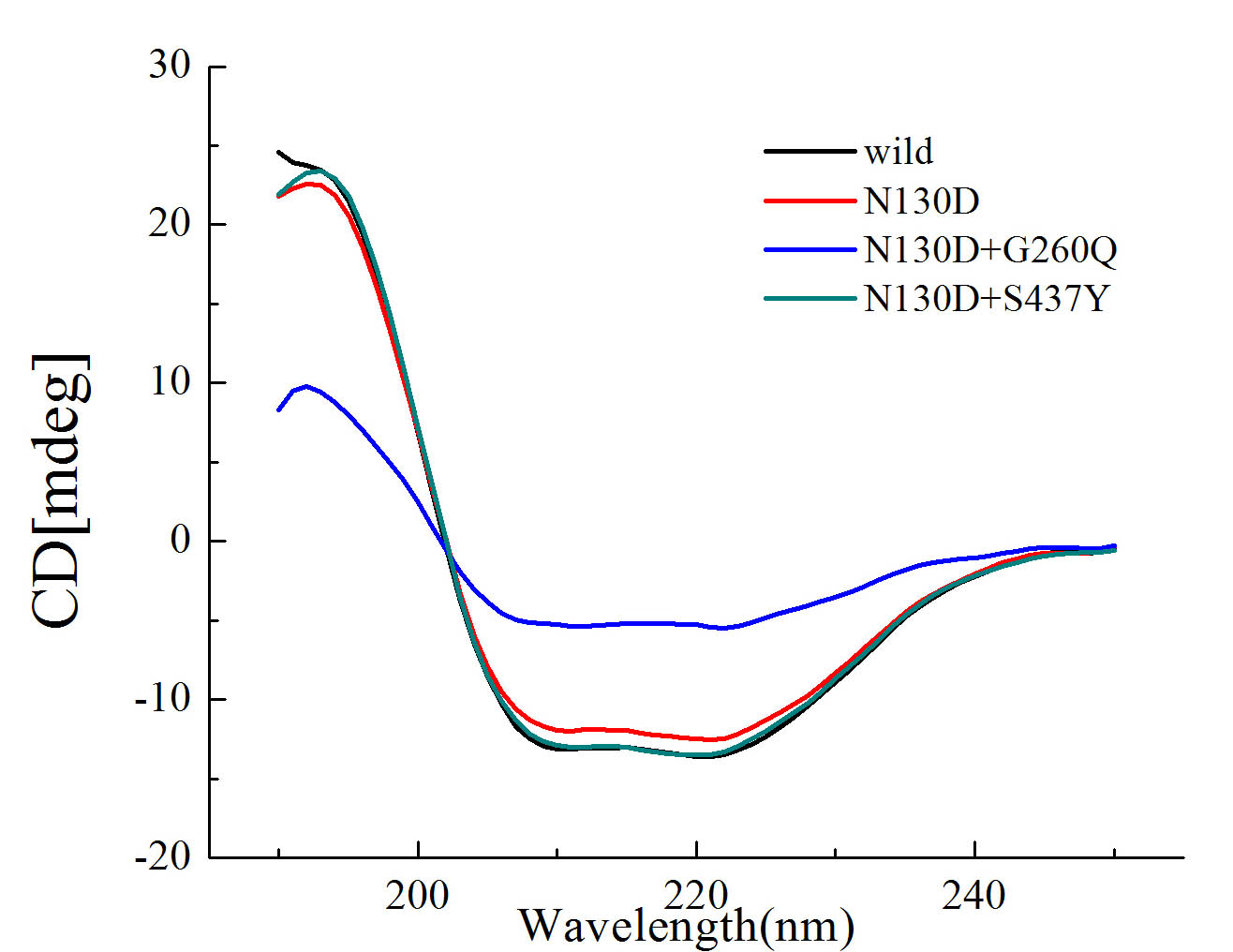

Supplement: Supplementary file 1 — Table S1. Saturation mutagenesis primers. Figure S1. Purification of wild-type and mutants. M: Marker, 1:wild, 2: N130D, 3: G260A, 4: S437T, 5: N130D/S437Y, 6: N130D/G260Q. Crude enzyme was loaded onto Ni-NTA resin to purify the protein, utilizing the His-tag encoded by pET-32a. SDS-PAGE analysis was performed on a 12% running gel and were visualized by Coomassie Brilliant Blue G-250 staining. Figure S2. Tm values of enzymes determined by differential scanning calorimetry. Figure S3. Circular dichroism of wild-type and mutants. (DOC 320 kb) [file 12896_2018_468_MOESM1_ESM.doc]
